# Depth-targeted intracortical microstroke by two-photon photothrombosis in rodent brain

**DOI:** 10.1117/1.NPh.9.2.021910

**Published:** 2022-03-16

**Authors:** Masahiro Fukuda, Takayoshi Matsumura, Toshio Suda, Hajime Hirase

**Affiliations:** a Kumamoto University, International Research Center for Medical Sciences, Kumamoto, Japan; b Duke-NUS Medical School, Signature Program in Neuroscience and Behavioral Disorders, Singapore; c Jichi Medical University, Division of Inflammation Research, Center for Molecular Medicine, Tochigi, Japan; d National University of Singapore, Cancer Science Institute of Singapore, Singapore; e University of Copenhagen, Center for Translational Neuromedicine, Faculty of Health and Life Sciences, Copenhagen, Denmark

**Keywords:** photothrombosis, multiphoton, mice, stroke, ischemia, cortex

## Abstract

**Significance:**

Photothrombosis is a widely used model of ischemic stroke in rodent experiments. In the photothrombosis model, the photosensitizer rose bengal (RB) is systemically introduced into the blood stream and activated by green light to induce aggregation of platelets that eventually cause vessel occlusion. Since the activation of RB is a one-photon phenomenon and the molecules in the illuminated area (light path) are subject to excitation, targeting of thrombosis is unspecific, especially in the depth dimension. We developed a photothrombosis protocol that can target a single vessel in the cortical parenchyma by two-photon excitation.

**Aim:**

We aim to induce a thrombotic stroke in the cortical parenchyma by two-photon activation of RB to confine photothrombosis within a vessel of a target depth.

**Approach:**

FITC-dextran is injected into the blood stream to visualize the cerebral blood flow in anesthetized adult mice with a cranial window. After a target vessel is chosen by two-photon imaging (950 nm), RB is injected into the blood stream. The scanning wavelength is changed to 720 nm, and photothrombosis is induced by scanning the target vessel.

**Results:**

Two-photon depth-targeted single-vessel photothrombosis was achieved with a success rate of 84.9%±1.7% and an irradiation duration of <80  s. Attempts without RB (i.e., only with FITC) did not result in photothrombosis at the excitation wavelength of 720 nm.

**Conclusions:**

We described a protocol that achieves depth-targeted single-vessel photothrombosis by two-photon excitation. Simultaneous imaging of blood flow in the targeted vessel using FITC dextran enabled the confirmation of vessel occlusion and prevention of excess irradiation that possibly induces unintended photodamage.

## 1 Introduction

Ischemic strokes occur at various levels of cerebral circulation ranging from massive middle cerebral artery occlusion to transient ministrokes that occur at single arterioles. Ischemia at the stroked sites causes neuronal death or irreversible damage that forms an infarct due to the lack of oxygen and energy substrates. Silent strokes typically occur in small arterioles and cause small-sized infarcts, resulting in little expressive symptoms. These so-called microstrokes occur in the cerebral parenchyma and remain undetected until multiple occurrences of microstrokes over a period integrate to develop into motor and/or cognitive deficits.^1^

Several models have been proposed to generate local embolic or thrombotic stroke in experimental animals.^2^^–^^4^ For instance, hundreds of microspheres of diameter 20 to 50  μm have been perfused into the upstream of cerebral circulation,^5^ e.g., the internal carotid artery, to induce embolic ischemic stroke in the downstream arterioles. Microsphere infusion imposes a fair amount of surgical burdens and inherently forms infarcts in multiple places. Since the locations of infarcts are somewhat arbitrary and sometimes ectopic, this method is not optimal for observing the impact of single-vessel occlusion by microscopic *in vivo* imaging.

Induction of thrombosis by optic means, photothrombosis,^6^ has been widely utilized by the stroke research community owing to the technique’s reproducibility and flexible target region selection. Photothrombosis is performed by introducing the photosensitizer rose bengal (RB) into the blood and exciting it by green light in the targeted area of the brain surface. RB excitation produces reactive oxygen species (ROS), which damages epithelial cells and promotes aggregation of platelet on the vessel wall that eventually occludes the vessel. Photothrombosis has the advantage of target area selection over other methods as the target region can be specified by guiding the excitation light illumination spot (for an example, Ref. 7). However, due to the nature of one-photon reaction, photothrombosis is not suited for targeting specific vessels inside the brain tissue because the superficial vessels including capillaries are also subjected to photothrombosis with even higher intensity light. Furthermore, excess exposure of high-intensity excitation light on the mouse cerebral cortex can penetrate into deeper structures, such as the hippocampus and striatum, and occlude off-target areas, making the interpretation of subsequent outcome unclear. Moreover, spot illumination of the thrombotic target area does not distinguish between arteries and veins.

Two-photon microscopy has been utilized widely in bioimaging due to its superior depth-penetration in the tissue and focus-specific fluorescence excitation.^8^ Taking advantage of this feature, multiphoton photothrombosis has been reported very recently by a group that employed fix-point irradiation.^9^ Here we introduce an alternative technique for depth-targeted photothrombosis (DTPT) that can readily be used on a standard two-photon microscope equipped with a resonant scanner. Using this method, we demonstrate photothrombosis in a single vessel or a group of vessels at a targeted depth of up to 300  μm. Remarkably, we show that photothrombosis can be induced at the cortical depth of 240 to 300  μm without unwanted occlusions above and below the target. These features are favorable for prospective microstroke research since inducing occlusion in individual targeted vessels in the brain parenchyma is considered to mimic silent stroke.

## 2 Materials and Methods

### 2.1 Subjects and Surgery

Adult C57BL/6 mice (2 to 6 months old, 20 to 30 g) of either sex were used. Mice were deeply anesthetized with isoflurane (5% for induction, 1.5% for maintenance). A craniotomy of ∼3  mm diameter was performed above the somatosensory cortex while the dura was left intact. Thereafter, a 3-mm diameter glass coverslip (No. 0; Matsunami, Japan) was placed and fixed with dental cement. A metallic head frame that fits to a MAG-3 head-stager holder (Narishige, Japan) was attached to the skull, and the remaining exposed skull was covered with dental cement.

The procedures involving animal care, surgery, and sample preparation were approved by the Danish Animal Experiments Inspectorate and the Animal Care and Use committee of Kumamoto University, Kumamoto, Japan. The animal study was overseen by the University of Copenhagen Institutional Animal Care and Use Committee.

### 2.2 In Vivo Two-Photon Depth-Targeted Photothrombosis

Two-photon imaging and DTPT was performed in anesthetized mice, using a Bergamo scope (Thorlabs, NJ, USA) equipped with a resonant scanner (8 kHz), a piezo Z drive, a 25×1.1 NA water immersion lens (CFI75 Apochromat 25XC W 1300; Nikon, Japan), and a Mai Tai eHP DeepSee laser (Spectra-Physics, CA, USA). Emission light was separated by a dichroic mirror (562 nm; Chroma, VT, USA) with bandpass filters 525/50 and 607/70  nm (both from Chroma) for the green and red channels, respectively. The microscope was operated using the ThorImage LS software version 4.0. Laser power at 950 and 720 nm was measured using a laser power meter (Fieldmate and PM10, Coherent, CA) before experiments every time.

DTPT was carried out as follows. First, to maximize the excitation efficiency, we adjusted the head-stage mount angle so that the craniotomy plane became parallel to the objective lens plane using the goniometer incorporated into the MAG-3 head-stage holder.^10^ Second, 300  μL of FITC-150k dextran (concentration: 20  mg/mL, Sigma-Aldrich, MO, USA) was injected intravenously (i.v.) via the retro-orbital route to label blood vessels. After identifying the target blood vessel using 950 nm excitation light, 100  μL of the photosensitizer RB (Sigma-Aldrich, MO, USA) was injected (i.v., RB concentration: 20  mg/mL in phosphate buffered saline)^11^ via a catheter placed in the tail vein. For multiple occlusion experiments, RB was injected every 20 min to maintain the RB concentration in the blood (up to three times). For targeting a single blood vessel at 240 to 300  μm, RB was injected only once.

The excitation light wavelength was then changed to 720 nm, and the focal depth was readjusted to the target vessel’s lumen using the least laser power possible (2.9 mW). Next, the scanned area was restricted by increasing the digital magnification to include only the targeted vessel. To start photothrombosis, the laser intensity at the exit of the objective lens was set to the necessary amount (31 mW for multiple occlusion experiments at 30 to 50  μm, 63 mW for targeting 240  μm, and 300 mW for targeting 300  μm). The selected area was subsequently scanned at a high frame rate (30 to 60 Hz, depending on the scanned area) while monitoring acquired images in real time. If equipped, the far-red detection channel should be switched off to protect PMT. Scanning was terminated when blood clots were formed.

### 2.3 Statistics

Comparisons of two sample means were assessed by t-test. Error bars of bar graphs represent the standard error of the mean. Box plots indicate the medians and 25th and 75th percentiles of the sample data. The whiskers of the box plots represent the shorter of the data range or the outlier limit. The outlier limits are defined as q3+1.5×(q3−q1) and q1−1.5×(q3−q1), where q1 and q3 are the 25th and 75th percentiles of the sample data.

## 3 Results

### 3.1 Repetitive Fast Scans in the Lumen Induces Two-Photon Photothrombosis

**Fig. 1:**
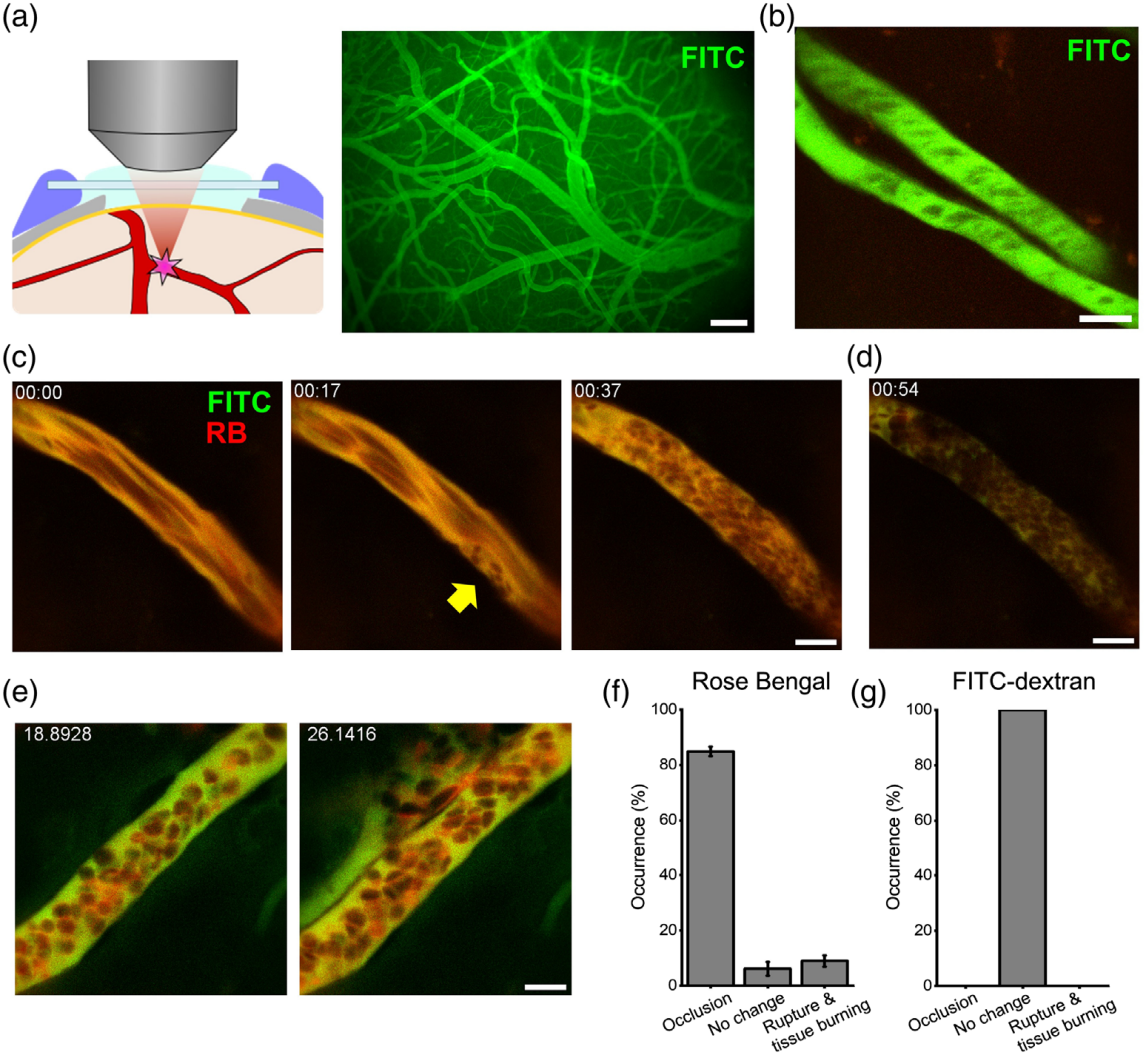
*In vivo* two-photon DTPT: (a) schematic illustration of DTPT. After visualization of blood vessels with FITC-dextran, a blood vessel containing RB is targeted by two-photon excitation. (b) Blood vessels to be targeted were chosen using FITC-dextran fluorescence. (c) Following RB injection, the excitation wavelength was switched to 720 nm. Imaging of the targeted blood vessel with 720 nm wavelength was performed until coagulation was formed. (d) Completion of occlusion could be distinguished by photobleaching of RB and FITC. (e) In a small number of cases, blood vessel rupture or tissue burning was observed. (f) The success rate of DTPT was 84.9%±1.7%, whereas 6.1%±2.5% of the cases were not occluded (no change) and 9.0%±2.1% of the cases had tissue burning/rupture (occlusion: 84/99, no change: 6/99, tissue burning/rupture 9/99 vessels, 4 mice). (g) Controls with FITC-150k dextran only did not show occlusion or tissue burning/rupture in 150 s (103 blood vessels, 3 mice). Scale bars: (a) 200  μm and (b)–(e): 10  μm. Occlusion of a single cortical vessel by DTPT. Scale bar 10  μm. (Video 1, MP4, 7 MB [URL: https://doi.org/10.1117/1.NPh.9.2.021910.1]).

To undertake the induction of photothrombosis by an ultrashort pulse infrared laser, we followed a recent paper that reported an enhanced multiphoton excitation of RB for wavelengths <750  nm.^12^ First, we visualized the cerebral vasculature by labeling the serum with FITC-dextran via retro-orbital i.v. injection and imaging through the cranial widow by a two-photon microscope in an anesthetized mouse [Fig. 1(a)]. A wavelength of 950 nm was used to excite FITC. Cerebral arterioles were identified by tracking the downstream of a descending blood flow in the penetrating arteries. A vessel of diameter 4 to 20  μm was chosen as a target and placed at the middle of the view field by adjusting the microscope stage. In selecting a target vessel, locations with large vessels lying above them were avoided. The scanned area was restricted to the target vessel by zooming into the vessel [Fig. 1(b)]. After injecting RB i.v. via a catheter placed in the tail vein, the wavelength was changed to 720 nm and the scanned depth (the Z axis focus) was readjusted to scan inside the lumen with a minimum laser intensity (2.9 mW) with a frame rate at 30 or 60 Hz. After setting the target, the laser power was set to 31 mW (at the exit of the objective lens). The PMT voltage and amplifier gain was set to low values to allow for simultaneous photothrombosis and vessel blood flow monitoring.

The blood flow of the subjected vessel was recognized from the suboptimal fluorescence of RB and FITC as acquired images are updated on the screen [Fig. 1(c), left]. Initially, platelets started to attach to the blood vessel wall [Fig. 1(c), middle]. Thereafter, platelets and red blood cells (RBCs) made a clot, and the blood flow was gradually compromised [Fig. 1(c), right; Video 1]. Occlusion of the vessel was observed by the appearance of stalled platelets and RBCs in the image. In addition, the blood-contained dyes quickly became bleached and after complete occlusion, which can be used as the sign of occlusion [Fig 1(d)]. Occlusion was induced with 720 nm irradiation of duration shorter than 80 s in the majority of cases [Fig. 1(f), 84/99 vessels, 4 mice], though we have observed failure of occlusion or rupture/tissue burning in a small number of attempts [Fig. 1(f)]. In contrast, the FITC-only control displayed neither occlusion nor attachment of platelets to the vessel walls even when they were irradiated for up to 150 s [Fig. 1(g)].

**Fig. 2:**
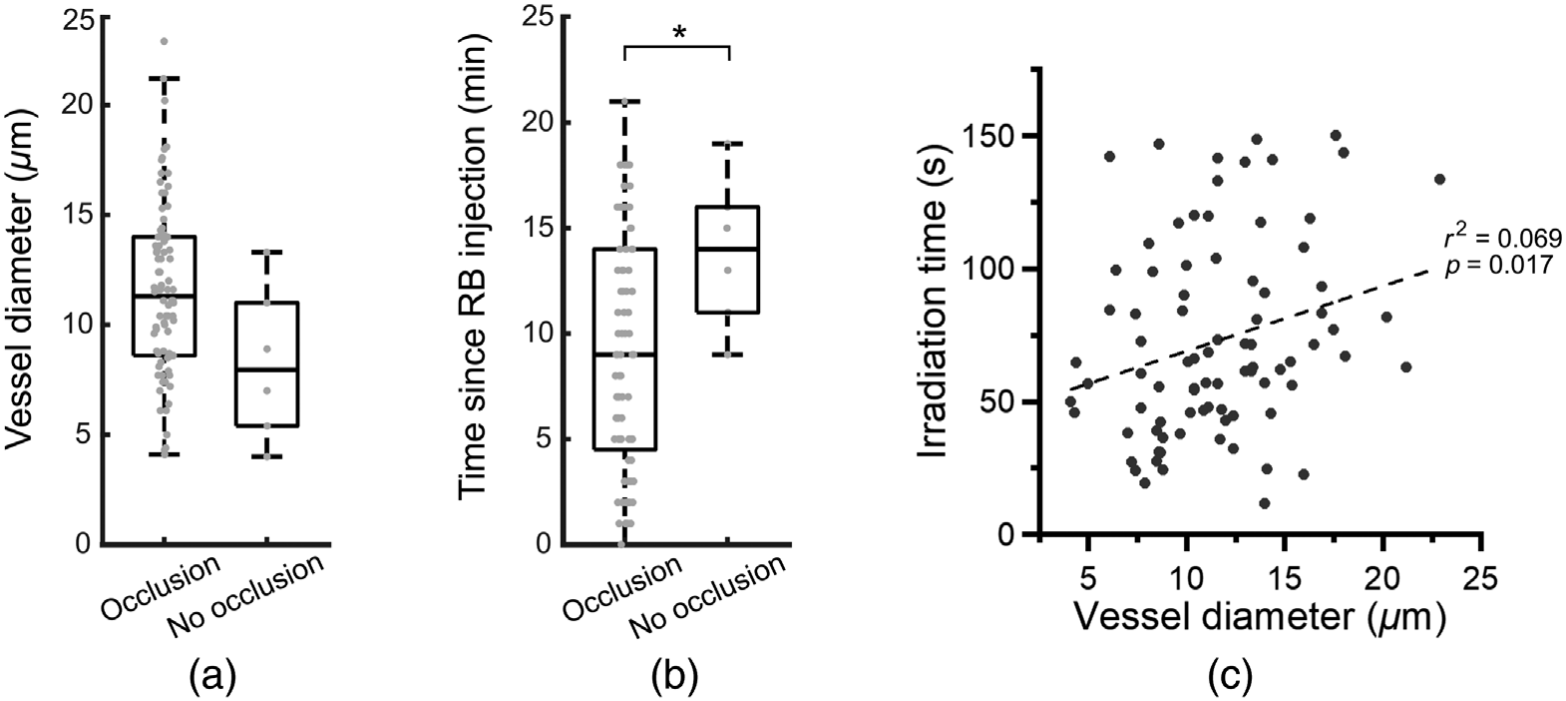
Parameters for successful DTPT: (a) distribution of vessel diameter for successfully occluded vessels versus not occluded vessels. (b) Distribution of time since RB injection for successfully occluded vessels versus not occluded vessels. *P<0.05. (c) Scatter plot showing the relationship between irradiation time and vessel diameter for successfully occluded vessels.

We did not find that the diameter of blood vessels affected the success of occlusion formation (occlusion: 11.58±0.43  μm (n=84), no change: 8.27±1.57  μm (n=6), P=0.069) [Fig. 2(a)]. We find that the success rate of occlusion formation by DTPT is affected by the time since the RB injection. The unsuccessful attempts occurred when significantly longer time passed after the RB injection than typical successful attempts [successful occlusion: 9.0±0.6  min (n=84), no occlusion: 13.8±1.6  min (n=6) since the RB injection. P=0.019] [Fig. 2(b)]. These results together with the FITC-only control [Fig. 1(g)] indicate that the concentration of RB inside the blood vessel is crucial for DTPT and 720-nm excitation laser reliably excites RB, most likely due to the relatively high excretion of blood RB to bile.^13^ We calculated the correlation between irradiation time and vessel diameter for successfully occluded vessels and found that they are only weakly correlated ($r^{2}=0.069$, p=0.017).

### 3.2 DTPT Occlusion is Confined to the Targeted Vessels

**Fig. 3:**
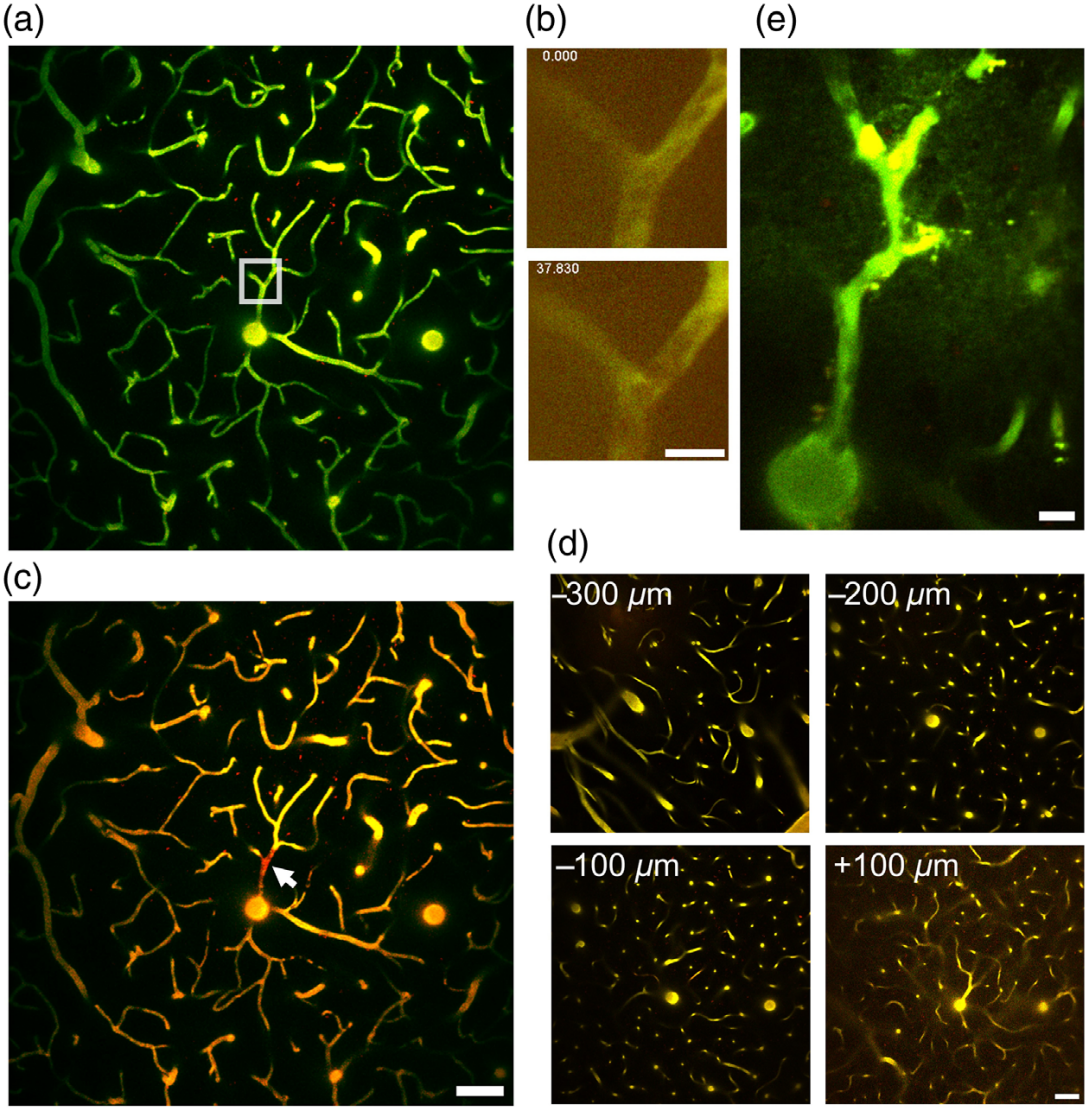
Off-target effect of DTPT is negligible: (a) the blood vessel located at 300  μm from the pia was targeted. (b) Occlusion was formed in 40 s using 300-mW 720-nm excitation laser. (c) After DTPT, the excitation was set to 950 nm, and coagulation was visualized. (d) Even using 300 mW excitation for DTPT, there was no occlusion/coagulation in areas 300, 200, 100  μm above and 100  μm below the target. (e) Extravasation of FITC-150k dextran and occlusion of peripheral blood vessels were observed three days after DTPT. Scale bars: (a), (c), (d) 50  μm and (b), (e) 10  μm. Occlusion of the vessel corresponding to (b). Scale bar 10  μm. (Video 2, MP4, 5 MB [URL: https://doi.org/10.1117/1.NPh.9.2.021910.2]). Depth stack of the cortical vasculature around the DTPT target site corresponding to this figure (Video 3, MP4, 12 MB [URL: https://doi.org/10.1117/1.NPh.9.2.021910.3]) and Fig. S1 in the Supplementary Material (upper panel, Video 4, MP4, 4 MB [URL: https://doi.org/10.1117/1.NPh.9.2.021910.4]) and (lower panel, Video 5, MP4, 5 MB [URL: https://doi.org/10.1117/1.NPh.9.2.021910.5]). Scale bar 50  μm.

The DTPT attempts that we have described thus far have been done in target planes 30 to 50  μm from the pial surface; next we perform DTPT targeting single blood vessels (φ=6.3 to 7.0  μm) located at the depths of 240 and 300  μm. In our attempts, one vessel per animal was subjected to DTPT. For the depth of 300  μm, we used the laser power 300 mW for the 720-nm irradiation in a mouse. Accordingly, the blood clot was formed within 40 s [Figs. 3(a) and 3(b), Video 2]. The clot was clearly visible with 950 nm excitation [Fig. 3(c), arrow]. In two mice, we performed DTPT with 63 mW irradiation at depth 240  μm. Targeted vessels were successfully occluded by ∼90-s irradiation (Fig. S1 in the Supplementary Material). Together, all three attempts of deep-vessel DTPT were successful.

The utility of DTPT becomes powerful when off-focus vessels are not occluded. To ensure that photothrombosis was confined to the targeted vessel, we took volumetric images and examined whether there are any blood clots in the capillaries 100, 200, and 300  μm above and 100  μm below the target plane shortly after DTPT (within 5 min) at a wavelength of 950 nm. As shown in a representative image in the result, we saw no obvious clots in the off-target planes [Fig. 3(d) and Videos 3, 4, and 5]. To examine the effect of selective occlusion, we observed the area 3 days later. We found that the peripheral blood vessels of the targeted plane were also occluded, and extravasation of FITC around the occluded blood vessels was discernible [Fig. 3(e)].

## 4 Discussion

Photothrombosis has been studied for over three decades^6^ and remains widely used for targeted induction of ischemic stroke owing to its superior reproducibility. Despite the widespread utility, photothrombosis has not been utilized for induction of microstrokes targeted specifically at a depth of the cerebral cortex until very recently. This is because conventional photothrombosis relies on one-photon excitation of RB: ROS is produced wherever the green photon hits RB; hence the formation of thrombosis is biased toward superficial vessels. On the other hand, multiphoton excitation occurs only at the focal spot, avoiding the production of ROS production outside the focal plane, and thereby enables depth-specific targeting. Indeed, Delafontaine-Martel et al. demonstrated that multiphoton occlusion is feasible by two-photon or three-photon excitation. In their study, two-photon photothrombosis was achievable in depths up to 200  μm from the pial surface, and significantly longer irradiation time (up to 300 s) was needed with an excitation wavelength of 1000 nm.^9^ In the current work, we further confirm that two-photon photothrombosis is feasible in the mouse brain. Notably, we find that <80-s irradiation is sufficient for inducing microvessel occlusion at a success rate >80% for vessels located in depths 30 to 50  μm. This accelerated two-photon photothrombosis was possible with a significantly shorter wavelength of 720 nm in the RB-injected mice but not in the FITC control mice.

The one-photon absorption spectrum of RB has a broad peak at around 560 nm, and a green light of range 530 to 580 nm is typically used for RB activation. Recently, Campaign and Knox^12^ measured the two-photon excitation spectrum of RB. According to this study, the two-photon-excited fluorescence increased by approximately threefold as the excitation wavelength shifts from 780 to 720 nm. Although the fluorescence emission does not necessarily correlate to ROS production, the demonstration of elevated molecule excitability at 720 nm prompts a future study to characterize ROS production around this wavelength. It is noteworthy that we could not observe discernible off-target photothrombosis even after targeting 240 to 300  μm with high laser power [Fig. 3(d); associated Videos 3, 4, and 5; and Fig. S1 in the Supplementary Material], indicative of focal spot-specific multiphoton excitation of RB with this wavelength. Another methodological point that we consider important for the induction of efficient photothrombosis is scanning the lumen of the target vessel and avoiding irradiating vessel walls (endothelial cells) as much as possible. This procedure, combined with simultaneous irradiation and imaging, is designed to ensure excitation of RB without damaging the vessel integrity. For this matter, it is imperative to perform DTPT shortly after RB administration, so the plasma retains sufficient RB levels.

Although not attempted in this study, the shortened time of photothrombosis induction makes it likely that this method is applicable to awake mice, which eliminates confounding factors associated with anesthetics^14^ and hence mimics human strokes more closely. Although photothrombosis of a single small-sized vessel would not impact the cognitive ability of the subjected mouse, we demonstrate that DTPT can be performed on multiple vessels, hence enabling scalable ischemic damage. A previous study has documented formation of a single-vessel clot using a kilohertz pulsed laser of pulse energy a few orders of magnitude more intense than a typical laser used for two-photon imaging.^15^ However, this method requires an intricate extra optics setup to produce the high-energy pulses. Our method works on a standard two-photon microscope without any hardware modifications; hence it appeals to the need for simplicity and versatility. It would be interesting to observe acute and chronic local perfusion changes after single-vessel DTPT and compare the outcome with the published results using high-energy pulses.^15^ The feasibility of vasculature imaging three days after DTPT [Fig. 3(e)] is encouraging for such a study.

Single-vessel photothrombosis has been performed on surface vessels,^16^^–^^20^ but the supposed off-target effects on deeper parenchymal vessels have not been thoroughly addressed. A clever application of one-photon photothrombosis is targeting penetrating vessels,^21^ thereby partially circumventing the issue of depth-axis-wide activation of RB. DTPT offers a distinct advantage over the conventional green light-induced photothrombosis using infrared light that penetrates deeper into the parenchyma. Hence, DTPT should be an effective means to evaluate the theoretical prediction of microstroke outcomes in various microcirculation configurations.^22^ Future applications of the DTPT include occlusion of subcortical regions such as the hippocampus, where two-photon or three-photon imaging from the cortical surface is possible.^10^^,^^23^^–^^25^ Our preliminary attempts suggest that this is feasible, although a thorough histological investigation of the cortical areas in the passage of the infrared beam should be performed to confirm target-specific induction of thrombotic stroke.

Another future utility of DTPT is to investigate the impact of single microvessel occlusion on the surrounding neuropil activity and inflammation. Astrocytes, a major glia subtype, have been reported to elicit aberrant activities after wide field photothrombosis.^26^^,^^27^ Chronic imaging ${\mathrm{Ca}}^{2+}$ activity or morphology will be a great resource for understanding how neuropils normalizes after the mild ischemic insult or how angiogenesis possibly occurs. Of note, we have recently described that adrenergic receptor antagonism accelerates neuronal activity recovery after wide field photothrombotic stroke^27^ or cortical spreading depression/depolarization.^28^ It would be interesting to examine how adrenergic receptor antagonism affects the homeostasis of the extracellular environment around a singly occluded vessel in light of the adrenergic receptor-dependent glymphatic system.^29^^,^^30^

## Code, Data, and Materials Availability

All materials used in the current procedure are available commercially. ThorImage LS is proprietary image acquisition software for Thorlab’s multiphoton microscopes.

## References

[1] Vermeer S. E. Longstreth W. T. Koudstaal P. J., “Silent brain infarcts: a systematic review,” Lancet. Neurol. 6(7), 611–619 (2007). 10.1016/S1474-4422(07)70170-9 17582361

[2] Durukan A. Tatlisumak T., “Acute ischemic stroke: overview of major experimental rodent models, pathophysiology, and therapy of focal cerebral ischemia,” Pharmacol. Biochem. Behav. 87(1), 179–197 (2007). PBBHAU 10.1016/j.pbb.2007.04.015 17521716

[r3] Fluri F. Schuhmann M. K. Kleinschnitz C., “Animal models of ischemic stroke and their application in clinical research,” Drug Des. Dev. Ther. 9, 3445–3454 (2015). 10.2147/DDDT.S56071 PMC4494187 26170628

[4] Sommer C. J., “Ischemic stroke: experimental models and reality,” Acta Neuropathol. 133(2), 245–261 (2017). ANPTAL 1432-0533 10.1007/s00401-017-1667-0 28064357 PMC5250659

[5] Kudo M. et al., “An animal model of cerebral infarction. Homologous blood clot emboli in rats,” Stroke 13(4), 505–508 (1982). SJCCA7 0039-2499 10.1161/01.STR.13.4.505 7101352

[6] Watson B. D. et al., “Induction of reproducible brain infarction by photochemically initiated thrombosis,” Ann. Neurol. 17(5), 497–504 (1985). 10.1002/ana.410170513 4004172

[7] Balbi M. et al., “Targeted ischemic stroke induction and mesoscopic imaging assessment of blood flow and ischemic depolarization in awake mice,” Neurophotonics 4(3), 035001 (2017). 10.1117/1.NPh.4.3.035001 28721356 PMC5512458

[8] Denk W. Strickler J. H. Webb W. W., “Two-photon laser scanning fluorescence microscopy,” Science 248(4951), 73–76 (1990). SCIEAS 0036-8075 10.1126/science.2321027 2321027

[9] Delafontaine-Martel P. et al., “Multiphoton excitation of rose bengal to induce capillary photo-thrombosis,” Proc. SPIE 11629, 116290G (2021). PSISDG 0277-786X 10.1117/12.2577868

[10] Fukuda M. Ozawa K. Hirase H., “Imaging living organisms,” in Imaging from Cells to Animals In Vivo, Barroso M. M. Intes X., Eds., pp. 295–306, CRC Press, Boca Raton, Florida (2020).

[11] Yardeni T. et al., “Retro-orbital injections in mice,” Lab Anim. 40(5), 155 (2011). 10.1038/laban0511-155 PMC3158461 21508954

[12] Campaign S. M. G. Knox W. H., “Increase in efficacy of near-infrared femtosecond micromachining in ophthalmic hydrogels with the addition of sodium fluorescein, rose bengal, and riboflavin,” Appl. Opt. 58(32), 8959 (2019). APOPAI 0003-6935 10.1364/AO.58.008959 31873678

[13] Nakazima T., “Experimental and clinical studies on dyes for excretory function test of liver,” Tohoku J. Exp. Med. 56, 87–94 (1952). 10.1620/tjem.56.87 13005492

[14] Seto A. et al., “Induction of ischemic stroke in awake freely moving mice reveals that isoflurane anesthesia can mask the benefits of a neuroprotection therapy,” Front. Neuroenergetics 6, 1 (2014). 10.3389/fnene.2014.00001 24765075 PMC3982055

[15] Nishimura N. et al., “Targeted insult to subsurface cortical blood vessels using ultrashort laser pulses: three models of stroke,” Nat. Methods 3(2), 99–108 (2006).1548-7091 10.1038/nmeth844 16432519

[16] Clark T. A. et al., “Artery targeted photothrombosis widens the vascular penumbra, instigates peri-infarct neovascularization and models forelimb impairments,” Sci. Rep. 9(1), 2323 (2019). SRCEC3 2045-2322 10.1038/s41598-019-39092-7 30787398 PMC6382883

[r17] Sunil S. et al., “Awake chronic mouse model of targeted pial vessel occlusion via photothrombosis,” Neurophotonics 7(1), 015005 (2020). 10.1117/1.NPh.7.1.015005 32042854 PMC6992450

[r18] Taylor Z. J. Shih A. Y., “Targeted occlusion of individual pial vessels of mouse cortex,” Bio-Protoc. 3(17), e897 (2013). 10.21769/BioProtoc.897 27547784 PMC4991877

[r19] Sigler A. Goroshkov A. Murphy T. H., “Hardware and methodology for targeting single brain arterioles for photothrombotic stroke on an upright microscope,” J. Neurosci. Methods 170(1), 35–44 (2008). JNMEDT 0165-0270 10.1016/j.jneumeth.2007.12.015 18289696

[20] Kazmi S. M. S. et al., “Three-dimensional mapping of oxygen tension in cortical arterioles before and after occlusion,” Biomed. Opt. Express 4(7), 1061–1073 (2013). BOEICL 2156-7085 10.1364/BOE.4.001061 23847732 PMC3704088

[21] Shih A. Y. et al., “The smallest stroke: occlusion of one penetrating vessel leads to infarction and a cognitive deficit,” Nat. Neurosci. 16(1), 55–63 (2013). NANEFN 1097-6256 10.1038/nn.3278 23242312 PMC3952571

[22] Schmid F. et al., “The severity of microstrokes depends on local vascular topology and baseline perfusion,” Elife 10, e60208 (2021). 10.7554/eLife.60208 34003107 PMC8421069

[23] Horton N. G. et al., “*In vivo* three-photon microscopy of subcortical structures within an intact mouse brain,” Nat. Photonics 7(3), 205–209 (2013). NPAHBY 1749-4885 10.1038/nphoton.2012.336 PMC3864872 24353743

[r24] Kawakami R. et al., “Visualizing hippocampal neurons with *in vivo* two-photon microscopy using a 1030 nm picosecond pulse laser,” Sci. Rep. 3, 1014 (2013). SRCEC3 2045-2322 10.1038/srep01014 23350026 PMC3553458

[25] Streich L. et al., “High-resolution structural and functional deep brain imaging using adaptive optics three-photon microscopy,” Nat. Methods 18(10), 1253–1258 (2021).1548-7091 10.1038/s41592-021-01257-6 34594033 PMC8490155

[26] Ding S. et al., “Photothrombosis ischemia stimulates a sustained astrocytic ${\mathrm{Ca}}^{2+}$ signaling *in vivo*,” Glia 57(7), 767–776 (2009). GLIAEJ 1098-1136 10.1002/glia.20804 18985731 PMC2697167

[27] Monai H. et al., “Adrenergic receptor antagonism induces neuroprotection and facilitates recovery from acute ischemic stroke,” Proc. Natl. Acad. Sci. U. S. A. 116(22), 11010–11019 (2019). 10.1073/pnas.1817347116 31097598 PMC6561179

[28] Monai H. et al., “Adrenergic inhibition facilitates normalization of extracellular potassium after cortical spreading depolarization,” Sci. Rep. 11(1), 8150 (2021). SRCEC3 2045-2322 10.1038/s41598-021-87609-w 33854148 PMC8047013

[29] Iliff J. J. et al., “A paravascular pathway facilitates CSF flow through the brain parenchyma and the clearance of interstitial solutes, including amyloid β,” Sci. Transl. Med. 4(147), 147ra111 (2012). STMCBQ 1946-6234 10.1126/scitranslmed.3003748 PMC3551275 22896675

[30] Xie L. et al., “Sleep drives metabolite clearance from the adult brain,” Science 342(6156), 373–377 (2013). SCIEAS 0036-8075 10.1126/science.1241224 24136970 PMC3880190

