# Supplementary material for: Depth-targeted intracortical microstroke by two-photon photothrombosis in rodent brain
Source: Neurophotonics. 2022 Mar 16;9(2):021910. doi: 10.1117/1.NPh.9.2.021910 (PMC8929553; doi:10.1117/1.NPh.9.2.021910)
Supplement: Supplementary file 1 [file NPh_009_021910_SD001.pdf]

Supplementary materials

Depth-targeted intracortical microstroke by two-photon photothrombosis in rodent brain

Masahiro Fukuda[a,b], Takayoshi Matsumura[c,d], Toshio Suda[a,d], Hajime Hirase[e]

a International Research Center for Medical Sciences, Kumamoto University, Kumamoto, Japan
b Signature Program in Neuroscience and Behavioral Disorders, Duke-NUS Medical School, Singapore, Singapore
c Division of Inflammation Research, Center for Molecular Medicine, Jichi Medical University, Tochigi, Japan
d Cancer Science Institute of Singapore, National University of Singapore, Singapore, Singapore
e Center for Translational Neuromedicine, Faculty of Health and Life Sciences, University of Copenhagen, Copenhagen, Denmark

Step-by-step protocol for deep vessel-targeted photothrombosis

Setup:

Two-photon microscope equipped with a resonant-galvo scanner. (e.g., Thorlabs B-Scope or Bergamo)
Pockels cell or other laser power adjustment devices.
ThorImage LS software (version 4.0)
Tunable ultrashort pulse laser (e.g. Spectra Physics Maitai eHP DeepSee)
Power meter (Coherent Fieldmaste with a PM10 sensor head)
MAG-3 (Narishige) mouse head-plate mount.
CP-1 or CP-2 head plate (Narishige)

Laser power calibration:

1. Before imaging, place the power meter sensor head facing the objective lens.
2. Park the scanner at the center position, or maximize the digital magnification to target the center of the power meter.
3. Calibrate the laser power by measuring the power at the exit of the objective lens while varying the Pockels cell's input voltage.

Depth-targeted photothrombosis:

Animal preparations

1. A mouse is deeply anesthetized (isoflurane 5% induction, 1.5% maintenance) and attached to a stereotaxic frame (SR-5M-HT, Narishige)
2. After shaving hair, the skull is exposed, and the periosteum is removed with a scalpel.
3. A head plate (CP-1 or CP-2, Narishige) is glued to the skull using Super-Bond (Sun Medical).
4. A cranial window is prepared using a high-speed dental drill. Dura is kept intact and the cranial window is sealed with a 3 mm diameter glass coverslip (No. 0, Matsunami).
5. Insert a catheter into the tail vein.

Animal fixation under objective lens and imaging preparation

6. The mouse is kept under stable anesthesia (1.5% isoflurane).
7. The mouse is rigidly mounted to a MAG-3 head plate mount, and placed under the two-photon microscope.
8. Set the laser wavelength to 950 nm.
9. Using the goniometers of the MAG-3, the head plate angle is adjusted to be parallel to the objective lens surface.

Performing DTPT

10. Administer FITC dextran (150k Da, 20 mg/mL in PBS, 300 μL) i.v. via retroorbital sinus to label blood vessels.
11. Acquire a volumetric image (e.g., XYZ: 500 x 500 x 400 μm) using laser power range up to 30 mW at the exit of the objective lens (cf. laser power calibration)
12. Select a target vessel from the volumetric image. Change the focal plane and magnification accordingly.
13. Restrict the area of scanning to contain the targeted vessel. (e.g. 30 x 30 μm)
14. Inject Rose Bengal (20 mg/mL in PBS, 100 μL) i.v. via the tail vein.
15. Set the laser wavelength to 720 nm.
16. There will be a small shift in focus, so re-adjust the focal depth and scan area using the lowest power at 720 nm (e.g., 2.9 mW).
17. Lower the photomultiplier tube (PMT) voltage and gain so that the scanning does not damage PMT or saturate the resultant imaging in the next step.
18. Scan the region of interest with a high laser power (60–300 mW for depths 240–300 μm) until the target becomes occluded.
19. If targeting multiple blood vessels, move the focal position to the next target and repeat Step 17–18.

After DTPT

20. Set the laser wavelength to 950 nm and restore PMT voltages.
21. Acquire a volumetric image as in Step 11 to ensure that occlusion occurred only at the targeted vessel.

Figure S1

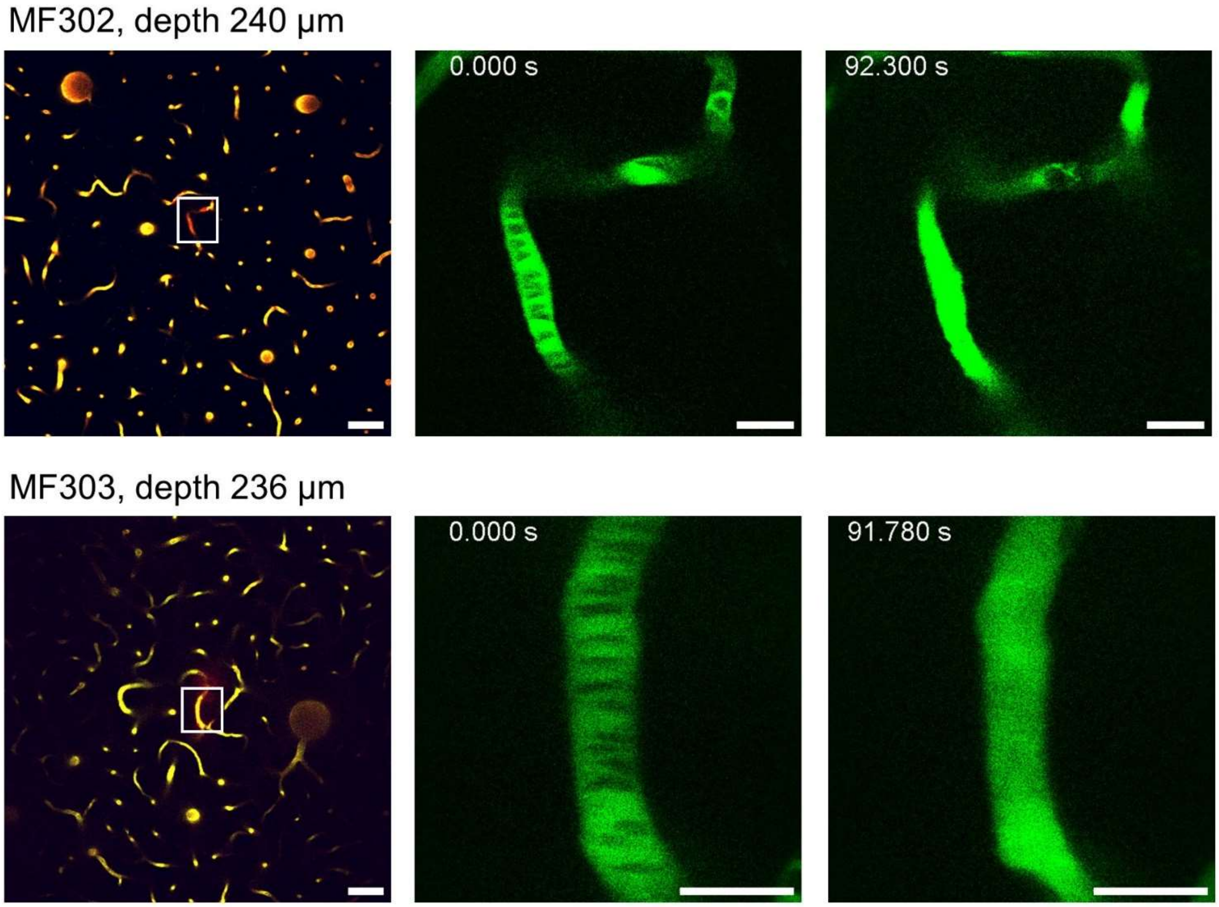

Fig. S1 Two examples of deep vessel DTPT. Two examples of deep-vessel DTPS are presented in each row. For both animals, the target depth is set to 240 µm. The left panels show the vasculature after successful DTPT at the target depth. Green and red channels correspond to FITC dextran 150k Da and Rose Bengal signals, respectively in the left panels. Green signal from FITC dextran is shown in middle and center panels. White boxes indicate the locations of target vessels. The middle panels show the targeted vessels at the start of DTPT. Red blood cells appear as black stripes in the vessels. The right panels show the occluded vessels after ~90 s of 720 nm, 63 mW irradiation. Scale bars: 50 µm (left panels), 10 µm (center and right panels).
